# A radiomics model based on magnetic resonance imaging to predict cytokeratin 7/19 expression and liver fluke infection of hepatocellular carcinoma

**DOI:** 10.1038/s41598-023-44773-5

**Published:** 2023-10-16

**Authors:** Jun-Qi Liu, Jing Wang, Xia-Ling Huang, Tian-Yi Liang, Xin Zhou, Shu-Tian Mo, Hai-Xiang Xie, Ke-Jian Yang, Guang-Zhi Zhu, Hao Su, Xi-Wen Liao, Li-Ling Long, Tao Peng

**Affiliations:** 1 Department of Hepatobiliary Surgery, The First Affiliated Hospital of Guangxi Medical University, Shuang Yong Rd. 6#, Nanning, 530021 Guangxi Zhuang Autonomous Region People’s Republic of China; 2 Guangxi Key Laboratory of Enhanced Recovery After Surgery for Gastrointestinal Cancer, Nanning, Guangxi Zhuang Autonomous Region People’s Republic of China; 3 Key Laboratory of Early Prevention & Treatment for Regional High Frequency Tumor (Guangxi Medical University), Ministry of Education, Nanning, Guangxi Zhuang Autonomous Region People’s Republic of China; 4 Department of Radiology, The First Affiliated Hospital of Guangxi Medical University, Nanning, Guangxi Zhuang Autonomous Region People’s Republic of China

**Keywords:** Hepatocellular carcinoma, Predictive markers, Prognostic markers

## Abstract

Hepatocellular carcinoma (HCC) is the most common type of primary liver cancer. HCC with liver fluke infection could harbor unique biological behaviors. This study was aimed at investigating radiomics features of HCC with liver fluke infection and establishing a model to predict the expression of cytokeratin 7 (CK7) and cytokeratin 19 (CK19) as well as prognosis at the same time. A total of 134 HCC patients were included. Gadoxetic acid-enhanced magnetic resonance imaging (MRI) images of all patients were acquired. Radiomics features of the tumor were extracted and then data dimensionality was reduced. The radiomics model was established to predict liver fluke infection and the radiomics score (Radscore) was calculated. There were 11 features in the four-phase combined model. The efficiency of the combined model increased significantly compared to each single-phase MRI model. Radscore was an independent predictor of liver fluke infection. It was also significantly different between different expression of CK7/ CK19. Meanwhile, liver fluke infection was associated with CK7/CK19 expression. A cut-off value was set up and all patients were divided into high risk and low risk groups of CK7/CK19 positive expression. Radscore was also an independent predictor of these two biomarkers. Overall survival (OS) and recurrence free survival (RFS) of negative liver fluke infection group were significantly better than the positive group. OS and RFS of negative CK7 and CK19 expression were also better, though not significantly. Positive liver fluke infection and CK19 expression prediction groups harbored significantly worse OS and RFS, survival of positive CK7 expression prediction was unsatisfying as well. A radiomics model was established to predict liver fluke infection among HCC patients. This model could also predict CK7 and CK19 expression. OS and RFS could be foreseen by this model at the same time.

## Introduction

Primary liver cancer (PLC) is one of the most common malignant tumors and its incidence is increasing greatly in recent years. Global cancer observatory (GLOBOCAN) showed that there were 840,000 new cases of liver cancer globally and 780,000 cases of liver cancer-related death in 2018, which ranked fourth among all cancers^1^. In its latest report in 2020, there were 900,000 new cases of liver cancer and 830,000 cases of death related to liver cancer. Its rank raised to third among cancer death^2^. Hepatocellular carcinoma (HCC) is the most common type of primary liver cancer which accounts to nearly 90% of all liver cancer types^3^. Sub-Saharan Africa and eastern Asia are areas with the most widespread cases of HCC^4^. The occurrence of HCC is the result of many factors including genetics and environment^5^. HCC with different backgrounds harbors unique biological behaviors, which can be closely linked to the progression of the patient’s condition and outcome of treatments^4, 6^.

Liver fluke infection can be one of these backgrounds, which are caused by food-borne parasites including *Clonorchis sinensis*, *Opisthorchis viverrini*, *Opisthorchis felineus* and *Fasciola hepatica*^7^. Liver fluke is a high-risk factor for cholangiocarcinoma and *Clonorchis sinensis* was identified as group 1 carcinogen in the report by IARC (International Agency for Research on Cancer) in 2012^8^. In the 4th report of neglected tropical diseases (NTDs) by World Health Organization (WHO), *Clonorchiasis* and *Opisthorchiasis* are emphasized for further prevention and control^9^. The main distribution areas of liver fluke infection are China and Southeast Asia. *Clonorchis sinensis* is the most common type of liver fluke distributed in China and provinces with high incidence of infection are Guangxi, Guangdong, Jilin and Heilongjiang^10–12^. Following liver fluke infection, the mechanical stimulation, components, and metabolites produced by the parasite induce chronic inflammation. This disrupts the normal physiological environment within the liver, leading to a series of processes including abnormal methylation, enhancing tumor heterogeneity, and simultaneously increasing the mutation burden^13^. Liver flukes can also inhibit apoptosis of HCC cells, thereby promoting tumor proliferation and metastasis. Therefore, for HCC patients with liver fluke infection, closer monitoring is required throughout the entire treatment period and more proactive adjuvant treatment strategies should be considered postoperatively^14, 15^.

Comprehensive evaluation of HCC is needed before further treatments as it could harbor different backgrounds^6^. An increasing number of methods can help the evaluation, including HBV virus copy number, glypican 3, heat shock protein 70, glutamine synthetase, staging systems such as Barcelona Clinic Liver Cancer (BCLC) and China Liver Cancer Staging (CNLC) as well as imaging examinations^3–16^. Ultrasonography (US), computed tomography (CT) and magnetic resonance imaging (MRI) have already been widely used during the management of various cancers^17–20^. Gadoxetic acid, a hepatocyte-specific contrast agent, has been applied to MRI and normal hepatocytes can take up this contrast agent^21^. For HCC, gadoxetic acid-enhanced MRI could help identify certain aggressive type, higher activity of proliferation and tendency of recurrence after transplantation^22–24^. However, the images contain a large amount of information, there is a need to further explore values of these images by new means. Radiomics was first introduced by Prof. Philippe Lambin in 2012 and it was aimed to convert information in the image into a huge number of quantitative features^25^. These features can help assessment of specific biomarkers, tumor surveillance and diagnosis, decision of treatment and evaluation of treatment sensitivity, thus assisting clinical decision-making^26, 27^.

CK7 and CK19 are expressed in hepatic progenitor cells between 4 and 10 weeks in embryonic stage. They turn negative when hepatic progenitor cells differentiate into hepatocytes^28^. CK7 and CK19 may possibly be expressed again when hepatocytes are under certain stimulation and tend to transform into hepatic progenitor cells^29^. Therefore, probably because of liver fluke infection, HCC tends to be CK7 or CK19 positive and harbors characteristics of cholangiocarcinoma, associated with faster proliferation rate and worse prognosis^30–32^.

**Figure 1:**
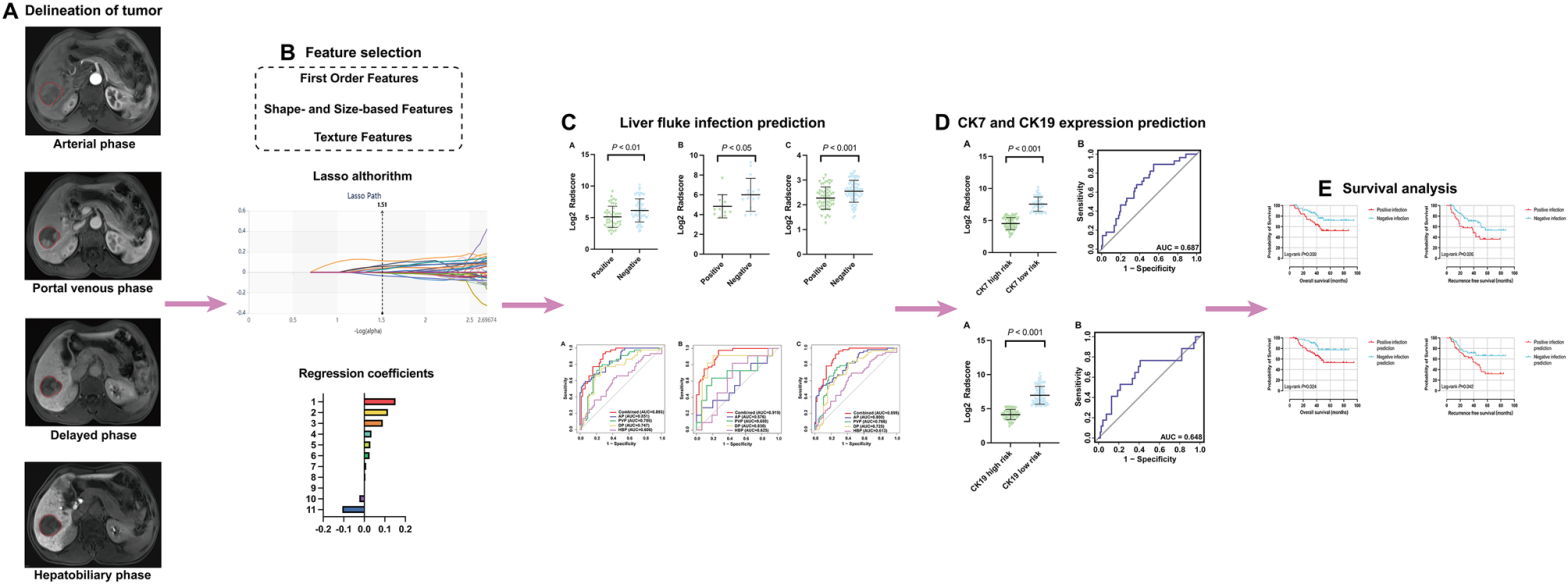
Flowchart of the whole study. (**A**) Region of interest was delineated layer by layer in the four stages of MRI images. (**B**) The extracted radiomic features were then went through data dimension reduction. (**C**) The radiomics model was established to predict liver fluke infection. The performance between four-phase combined model and single-phase model was compared. (**D**) The radiomics model was used to predict CK7 and CK19 expression. (**E**) Finally, Kaplan–Meier survival analysis was carried out.

Currently, confirmation of CK7 and CK19 expression relies on biopsy before operation or pathology after operation^33^. However, there are few studies about preoperative prediction of CK7 and CK19 in HCC based on gadoxetic acid-enhanced MRI by radiomics, especially in HCC with different backgrounds of liver fluke infection. Moreover, as far as we know, there are also very few studies focused on radiomics model predicting multiple indicators, like liver fluke, CK7 and CK19 in our study, at the same time and using the model to further predict overall survival and recurrence free survival. For countries like China where rates of both liver fluke infection and liver cancer remain high, it is necessary for a more comprehensive estimation of HCC features. In this study, we are aimed at investigating radiomics features of HCC with liver fluke infection and establishing a model to predict expression of CK7 and CK19 at the same time. Also, survival analysis is carried out between different groups of liver fluke infection, CK7 and CK19 expression, as well as radiomics predicting groups of these indicators (Fig. 1).

## Methods

### Patients

The cohort in this study was selected from HCC patients who underwent hepatectomy from March 2015 to June 2020 in the First Affiliated Hospital of Guangxi Medical University. Pathological examination was carried out immediately after the surgery. All specimens were preserved in formalin solution and then underwent pathological examination. The whole process was performed in accordance with Guidelines for the Diagnosis and Treatment of Hepatocellular Carcinoma^34^.

The diagnosis of liver fluke infection was carried out strictly according to the Diagnostic Criteria for Clonorchiasis (WS309-2009)^35^. The ELISA test was carried out according to the following steps: (1) Add the sample: Add 5 μL of the test serum sample into 500 μL of dilution buffer, mix thoroughly. Add dilution buffer to the positive and negative control groups according to the label instructions and mix thoroughly until dissolved. In the antigen pre-coated plate, set up 2 wells for the blank group with 100 μL of dilution buffer, 2 wells for the negative control group with 100 μL of negative control sample, 2 wells for the positive control group with 100 μL of positive control sample, and 5 wells for the test group, each receiving 100μL of diluted serum sample. Place the reaction system in an incubator at 37 °C for 1 h, remove all liquid from the wells, wash with washing solution 3 times, each for 1 min, and finally, after the last wash, air-dry the reaction plate. (2) Add the binding substance: Except for the blank group, add 100 μL of Horseradish Peroxidase (HRP)-conjugated binding substance to each well and incubate in an incubator at 37 °C for 1 h. Remove all liquid from the wells, wash with washing solution 3 times, each for 1 min, and finally, after the last wash, air-dry the reaction plate. (3) Add substrate solution: Add 100 μL of substrate solution to each well and incubate in the dark at 37 °C for 30 min. (4) Terminate the reaction and detection: Add 100 μL of stop solution to each well, detect the optical density (OD) at a wavelength of 450 nm using an enzyme-linked immunosorbent assay (ELISA) reader. If (sample OD value − blank control OD value)/(negative control OD value − blank control OD value) ≥ 2.1, it is considered as positive.

Liver fluke infection was considered as positive if eggs were found in fecal examination or liver flukes were found during surgery. Liver fluke infection was considered as negative when the following were satisfied: (1) these two examinations were negative, (2) ELISA (enzyme-linked immunosorbent assay) test was negative, (3) no characteristic images under B-mode ultrasonography (bile duct dilatation and wall thickening). The inclusion criteria were as follows: (1) patients were treatment-naïve when they were admitted to our hospital, (2) diagnosis of HCC was confirmed by pathological examination after the surgery, (3) patients received gadoxetic acid-enhanced MRI no more than 1 month before surgery. The exclusion criteria: (1) patients received transcatheter arterial chemoembolization or radiofrequency/microwave ablation before the MRI examination, (2) combined hepatocellular carcinoma and cholangiocarcinoma (cHCC-ICC) or intrahepatic cholangiocarcinoma carcinoma (ICC) was confirmed by pathological examination, (3) no stool examination to find liver fluke eggs, (4) either ELISA test or B-type ultrasonography was not performed during hospitalization if eggs were not found in fecal examination and liver flukes were not found during surgery.

Patient enrollment and data collection were completed between July 2021 and December 2022. Private information of all patients was censored. This study has passed the relevant ethics review of our institution (approval number: 2021 KY-E-095) and was carried out strictly according to Declaration of Helsinki.

### MRI image acquisition

All patients enrolled underwent plain and enhanced MRI examinations, which were all completed by a Siemens Verio 3.0 T magnetic resonance imaging scanner. This system worked with a 12-channel phased array coil. Volume interpolated body examination (VIBE) T1WI was performed with repetition time (TR) 3.9 ms, echo time (TE) 1.4 ms, flip angle 15°, field of view (FOV) 350 mm, matrix size 168 × 320 and slice thickness 4.5 mm. Parameters of T2WI were TR 2930 ms, TE 189 ms, FOV 400 mm and slice thickness 6 mm. For diffusion-weighted imaging (DWI), it was set with TR 9000 ms, TE 66 ms, FOV 420 mm and slice thickness 6 mm. Gadoxetic acid (Primovist, Bayer, Germany) was injected into the cubital vein at a rate of 2 mL/s and scans of arterial phase (AP), portal venous phase (PVP), delayed phase (DP) and hepatobiliary phase (HBP) were performed at 14 s, 40 s, 120 s and 20 min after the injection, respectively.

### Volume of interest

**Figure 2:**
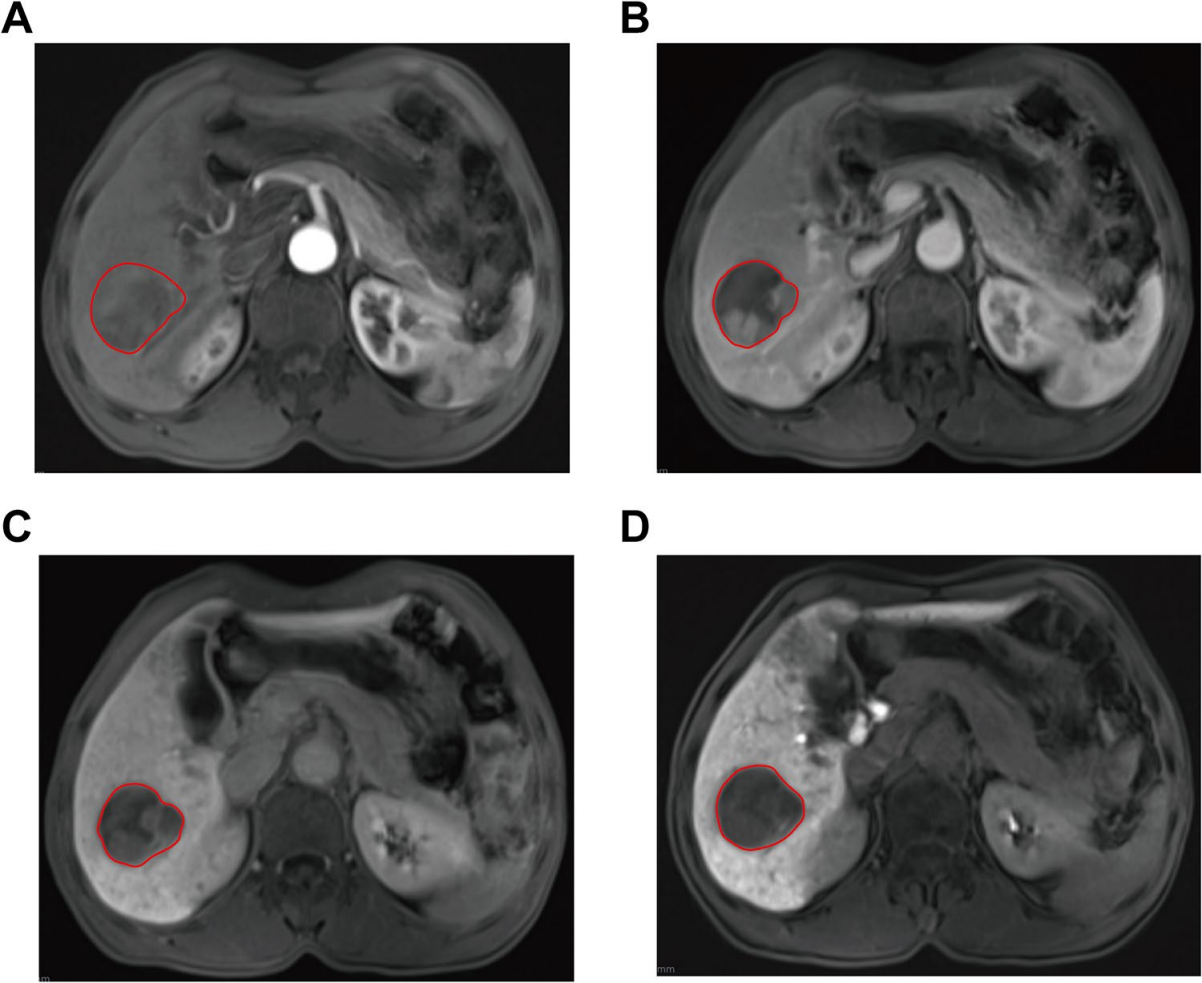
MRI images of arterial phase (**A**), portal venous phase (**B**), delayed phase (**C**) and hepatobiliary phase (**D**) of a 59-year-old male patient and delineation of region of interest.

MRI images of AP, PVP, DP and HBP were all acquired from the Picture Archiving and Communication Systems (PACS) and then uploaded to the Big Data Artificial Intelligence Radiomics Analysis System (Huiying Medical Technology, China). Region of interest was delineated layer by layer in the four stages of MRI and then fine-tuned to include the entire tumor lesion as much as possible (Fig. 2). It was carried out by a junior radiologist with 5 years of experience. Then, the delineation was validated by a radiologist with over 20 years of experience and was further checked by a radiologist with over 30 years of experience. Due to the subtle difference among images of the four phases of enhanced MRI, we carefully delineated the tumor boundary in each phase and took care to exclude the surrounding cancerous tissue. To further validate the accuracy of image delineation, we randomly selected 15 patients for a second round of MRI four-phase image delineation verification before further analysis. Volume of interest, a 3D model, was further constructed.

### Radiomic features and model construction

All patients were randomly divided into training group and validation group by cross validation of fivefold. The extracted radiomic features were went through Z-score normalization. VarianceThreshold, SelectKBest and the least absolute shrinkage (LASSO) were further used to reduce data dimension. There was a coefficient corresponding to each feature in the radiomics model, which was based on the two-distribution logistic regression formula. Radscore could be calculated accordingly. The corresponding *P* value in the logistic regression formula was defined as Risk score in this study.

The model performance was evaluated by supervised learning support vector machines (SVM) and K-nearest neighbor (KNN) classifiers. The performance of AP, PVP, DP, HBP and four-phase combined model was assessed in training group, validation group and the whole group. Receiver operating characteristic (ROC) curves were plotted and area under curve (AUC), sensitivity and specificity were calculated for each model. Delong test was performed to compare AUC values between ROC curves. Each clinical factor as well as Radscore were included in univariate and multivariate logistic regression analyses to test whether they were predictors of liver fluke infection.

### Construction of CK7 and CK19 prediction models

The expression of CK7 and CK19 was determined by pathological reports acquired from the PACS system. CK7 and CK19 expression was considered as positive (when ≥ 5% of tumor cells were positive) or negative (when < 5% of tumor cells were positive) by two pathologists who didn’t get access to medical history of the patient^36^.

Radscore was compared between groups of different CK7 and CK19 expression. ROC curve was plotted for each significant group. Clinical parameters were also compared. Univariate and multivariate statistical analyses were carried out to detect if any of clinical parameters and Radscore was predictor of CK7 or CK19 expression. The cut-off value was determined based on the Youden index and cases were classified into high-risk and low-risk groups.

### Survival analysis

All patients enrolled in this study were followed up according to their medical records and by telephone after the surgery. Recurrence of the tumor was confirmed through specific features in imaging examinations, including CT, MRI and ultrasound, when tumor appeared in the remaining liver, or there were distant organ and lymph node metastases. Deadline of the follow-up was Dec. 31, 2022.

### Statistical analysis

Independent *t* test or Mann Whitney *U* test was carried out for comparison between groups of continuous variables and chi-square test was for categorical variables. ROC curves were plotted by “pROC” package and forest plots were plotted by “tidyverse”, “ggplot2”, “survival” and “scales” in R project (https://www.r-project.org/). Scatter plots, bar plots and survival curves were completed by GraphPad Prism 9 (GraphPad Software, San Diego, CA, USA). All statistical analyses were performed by SPSS v.25.0 software (IBM, Chicago, IL, USA) and *P* value < 0.05 was considered statistically significant.

### Ethical statement

This study was approved by ethics review board of the First Affiliated Hospital of Guangxi Medical University (approval number: 2021 KY-E-095) and was carried out strictly according to Declaration of Helsinki. Since this was a retrospective study, written informed consent was waived by ethics review board of the First Affiliated Hospital of Guangxi Medical University and private information of all patients was censored.

## Results

### Clinical factors

Firstly, a total of 344 HCC patients who underwent both MRI examination and hepatectomy were collected. Following exclusion criteria, 65 cases were excluded due to the lack of preoperative MRI examination. In addition, 4 cases received interventional therapy prior to MRI examination, 6 cases underwent radiofrequency/microwave ablation, 123 cases did not receive stool examination for liver fluke eggs, and 12 cases did not receive liver fluke ELISA examination. Finally, there were altogether 134 cases enrolled in this study, and the level of ALT between groups of positive and negative liver fluke infection was significantly different (*P* = 0.019) (Table 1). There was no significant difference of all clinical parameters between training and validation groups (Table 2).

**Table 1:** Clinical parameters of patients with positive and negative liver fluke infection.

	Liver fluke infection positive (n = 55)	Liver fluke infection negative (n = 79)	*P* values
Gender			0.164
Male	51	67	
Female	4	12	
Age (years)			0.513
> 50	24	39	
≤ 50	31	40	
Hypertension			0.830
Yes	4	5	
No	51	74	
Diabetes			0.873
Yes	4	4	
No	51	75	
Total bilirubin (μmol/L)			0.946
> 20.5	10	14	
≤ 20.5	45	65	
Direct bilirubin (μmol/L)			0.233
> 6.8	12	11	
≤ 6.8	43	68	
ALT (U/L)			0.019
> 45	27	23	
≤ 45	28	56	
AST (U/L)			0.122
> 40	29	31	
≤ 40	26	48	
HBV infection			0.377
Yes	45	69	
No	10	10	
AFP (ug/L)			0.197
≥ 400	15	30	
< 400	40	49	
Maximum tumor diameter (cm)			0.462
< 3	17	30	
3 ~ 5	20	21	
> 5	18	28	
BCLC stage			0.109
A	31	58	
B	18	17	
C	6	4	

*ALT* alanine aminotransferase, *AST* aspartate aminotransferase, *HBV* hepatitis B virus, *BCLC* Barcelona clinic liver cancer.

**Table 2:** Clinical parameters of the training group and the validation group.

	Training group (n = 108)	Validation group (n = 26)	*P* values
Liver fluke infection			0.884
Positive	44	11	
Negative	64	15	
Gender			0.347
Male	97	21	
Female	11	5	
Age (years)			0.513
> 50	24	39	
≤ 50	31	40	
Hypertension			0.830
Yes	8	1	
No	100	25	
Diabetes			0.332
Yes	8	0	
No	100	26	
Total bilirubin (μmol/L)			0.929
> 20.5	20	4	
≤ 20.5	88	22	
Direct bilirubin (μmol/L)			1.000
> 6.8	19	4	
≤ 6.8	89	22	
ALT (U/L)			0.222
> 45	43	7	
≤ 45	65	19	
AST (U/L)			0.140
> 40	45	15	
≤ 40	63	11	
HBV infection			0.704
Yes	93	21	
No	15	5	
AFP (ug/L)			
≥ 400	37	8	0.735
< 400	71	18	
Maximum tumor diameter (cm)			0.367
< 3	36	11	
3 ~ 5	36	5	
> 5	36	10	
BCLC stage			0.829
A	73	16	
B	27	8	
C	8	2	

*ALT* alanine aminotransferase, *AST* aspartate aminotransferase, *HBV* hepatitis B virus, *BCLC* Barcelona clinic liver cancer.

### Radiomics features extraction and screening

**Figure 3:**
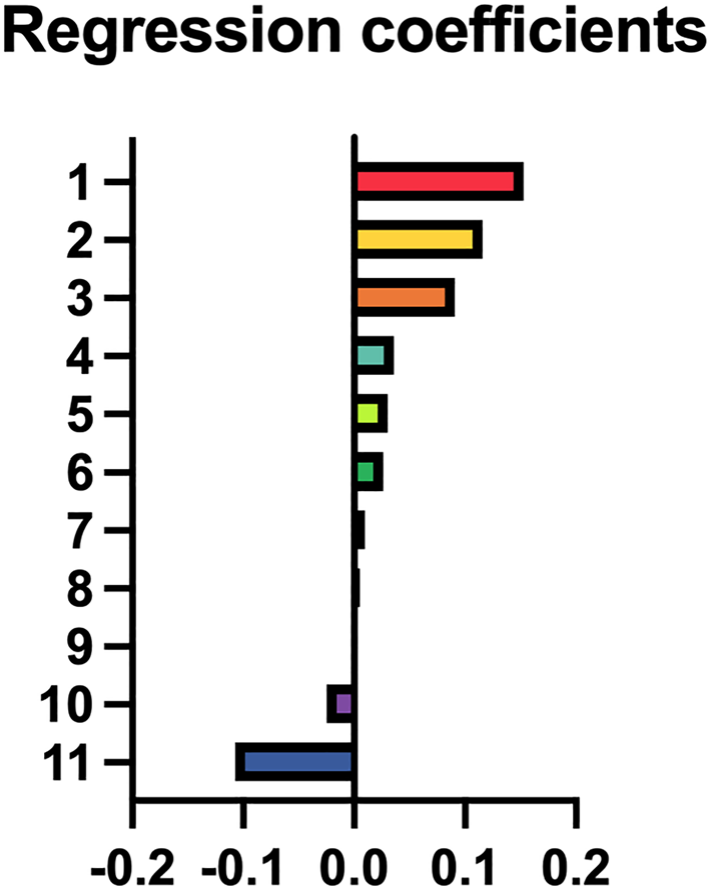
Corresponding regression coefficients of the features in the combined radiomics model, specific names and coefficients of the model is shown in Supplementary Table 1.

Through the radiomics analysis system, 1409 features were extracted from each of AP, PVP, DP and HBP images and there were 5636 features in the combined model. The features with variance values greater than 0.8 were screened by the VarianceThreshold and those with p < 0.05 were selected by the SelectKBest. After the LASSO algorithm to reduce data dimensionality, there were 16 features in the AP model, 5 features in the PVP model, 5 features in the DP model and 11 features in the combined model. The features and their regression coefficients in the combined model are shown in Fig. 3 (Supplementary Table 1).

### Establishment of radiomics model and performance evaluation

**Figure 4:**
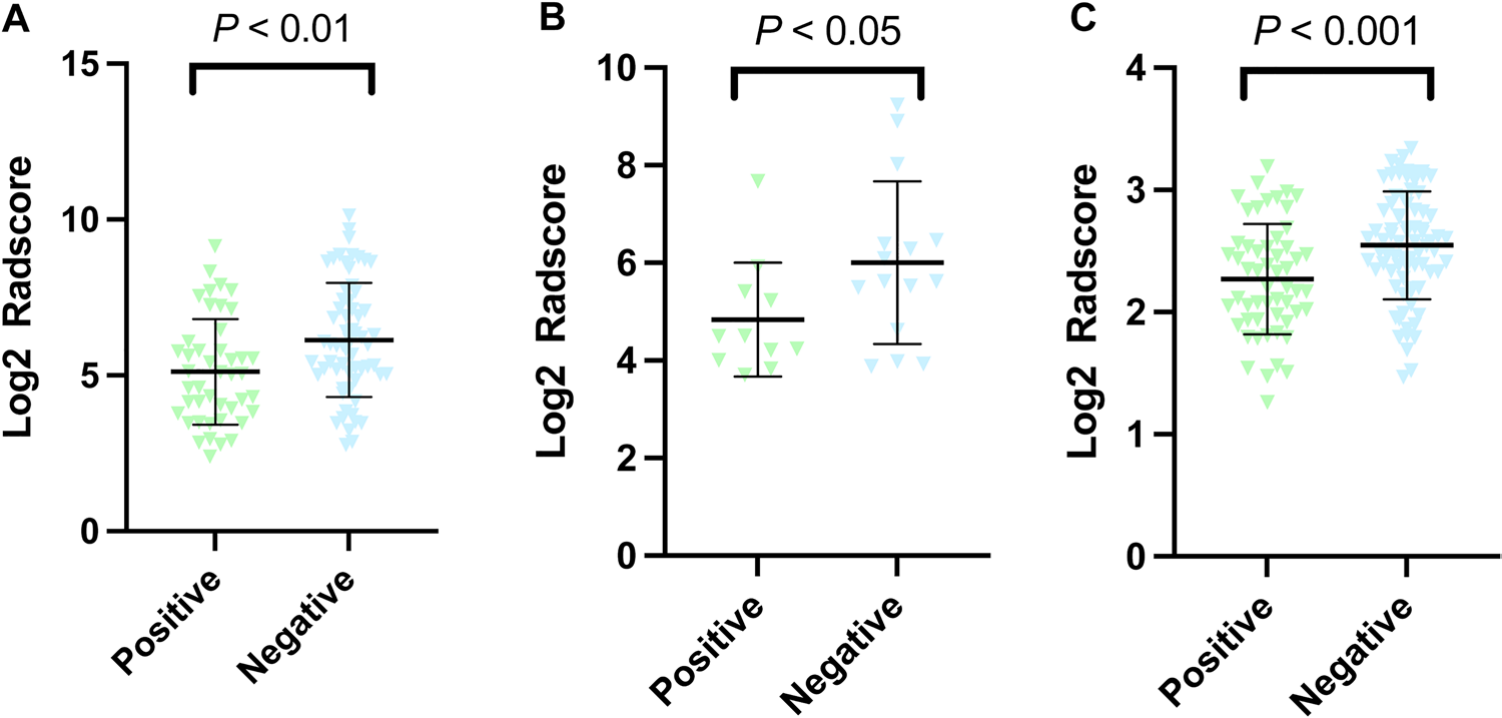
Radscore of patients with positive and negative liver fluke infection in training group, validation group and the whole group. (**A**) Training group, (**B**) Validation group, (**C**) Whole group.

**Figure 5:**
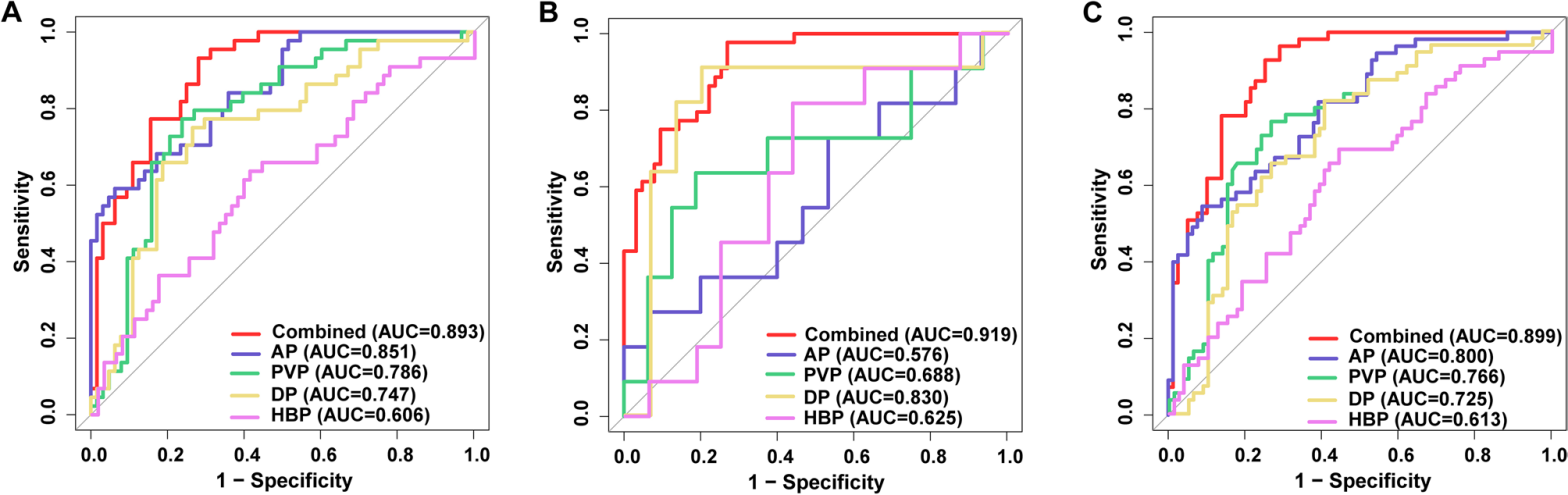
ROC curves of each single-phase model and combined model predicting liver fluke infection in training group, validation group and the whole group. (**A**) Training group, (**B**) Validation group, (**C**) Whole group. *ROC* receiver operating characteristic.

Radscores of the combined model between different liver fluke infection status were all statistically different in all three groups (Fig. 4). The evaluation of all models was performed by SVM and KNN classifiers in training, validation and the whole groups. The efficiency of each model was evaluated by AUC, specificity and sensitivity (Supplementary Table 2). ROC curves of all models predicting liver fluke infection were plotted in all three groups and the performance of the combined model was the best (Fig. 5). Delong's test was carried out to compare AP, PVP, DP, HBP and the combined models in training group, control group and the whole group. The prediction efficiency of the combined model increased significantly compared to AP, PVP, DP and HBP models (Supplementary Table 3).

**Figure 6:**
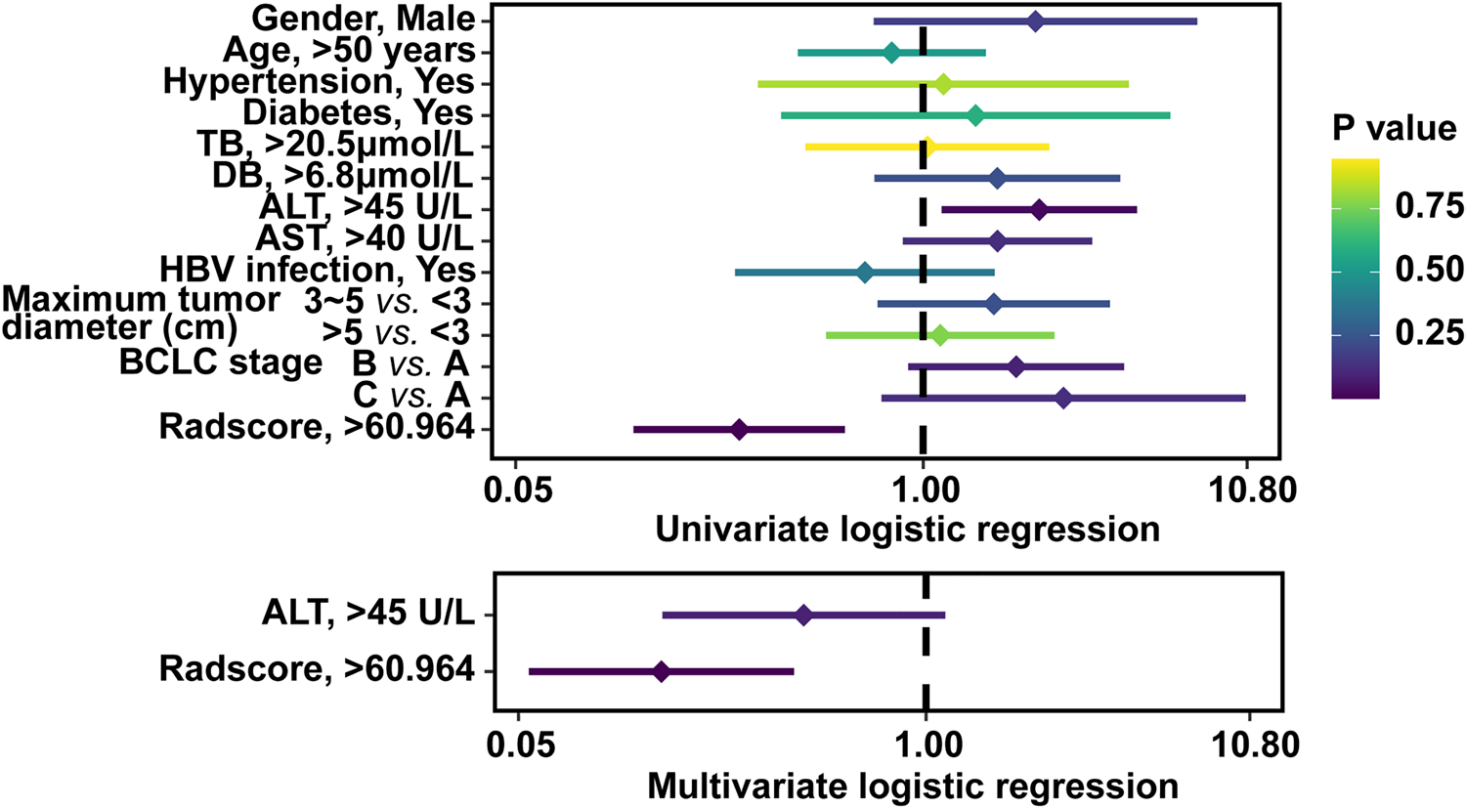
Univariate and multivariate logistic regression of clinical parameters and radiomics score for predicting liver fluke infection.

Each clinical parameter and Radscore were included in univariate and multivariate logistic regression to test whether they were independent predictors of liver fluke infection. Univariate logistic regression analysis showed that ALT level (*P* = 0.02, OR 2.348) and Radscore (*P* = 0.001, OR 0.259) could predict liver fluke infection and Radscore was an independent predictor (*P* = 0.001, OR 0.143, 95 CI% 0.054–0.379) (Fig. 6) (Supplementary Table 4).

### Survival analysis of different liver fluke infection and its prediction groups

**Figure 7:**
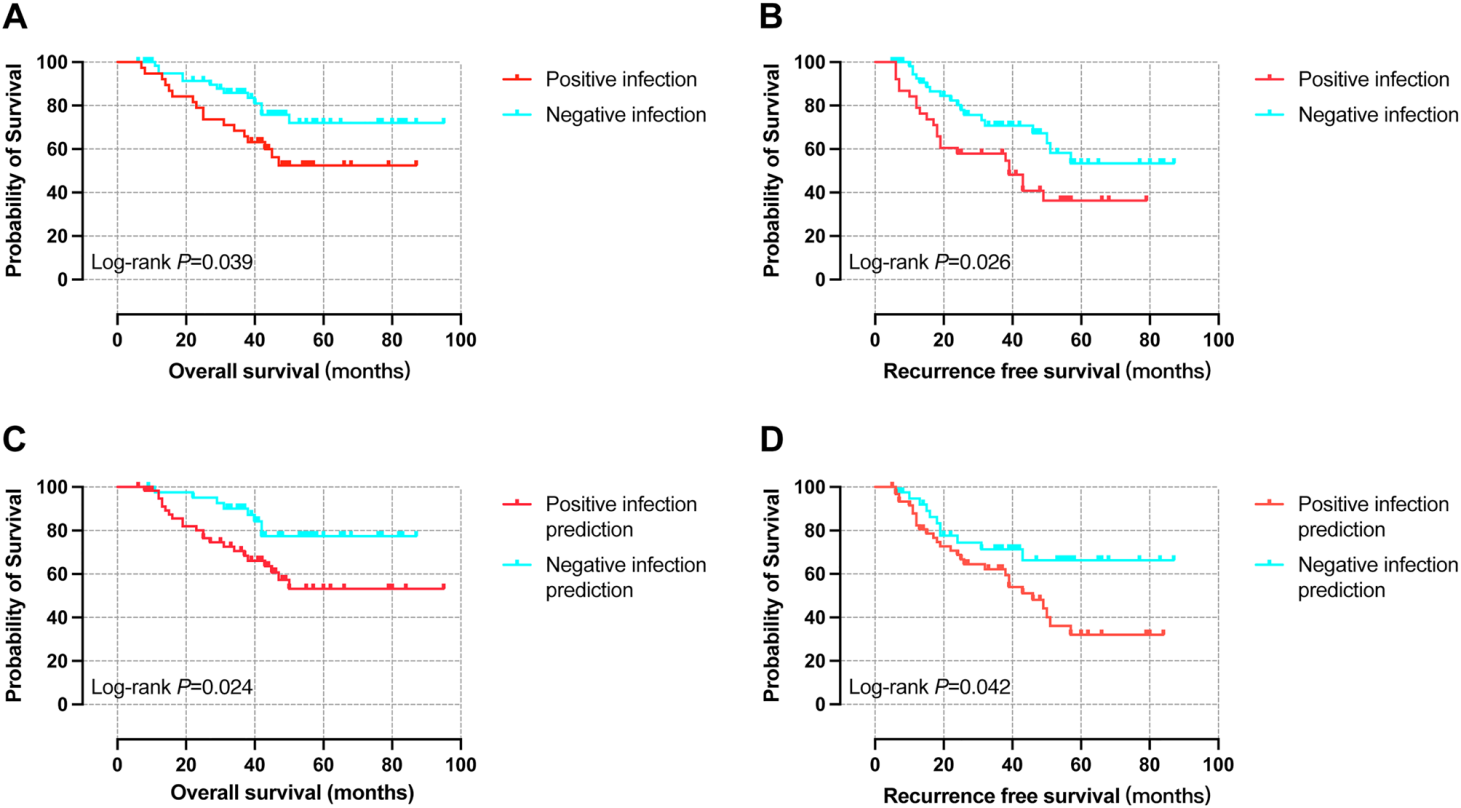
Kaplan–Meier survival analysis of different liver fluke infection and its prediction groups. (**A**) OS curves of positive and negative liver fluke infection, (**B**) RFS curves of positive and negative liver fluke infection, (**C**) OS curves of positive and negative liver fluke infection prediction, (**D**) RFS curves of positive and negative liver fluke infection prediction. *OS* overall survival, *RFS* recurrence-free survival.

There were 103 patients who were followed up in the survival analysis. 30 patients (29.1%) deceased and tumor recurrence happened in 40 patients (38.9%) after the liver resection. Overall survival (OS) of the negative liver fluke infection group was better than the positive group (P = 0.039), recurrence free survival was also more satisfying (P = 0.026), both reaching statistically significant. At the same time, OS and RFS of negative infection prediction group were significantly better than positive prediction group (P = 0.024 and P = 0.042, respectively) (Fig. 7).

The survival rates of negative liver fluke infection group were higher than those in positive group (24 months: 91.4% *vs.* 78.9%, 36 months: 85.9% *vs.* 68.4%, 60 months: 72.0% *vs.* 52.5%). Similar results were found in the comparation of no recurrence rates between negative and positive liver fluke infection groups (24-months: 80.2% *vs.* 57.9%, 36 months: 70.8% *vs.* 54.7%, 60 months: 53.4% *vs.* 36.3%). The survival rates of negative infection prediction group were also higher than those of positive prediction group (24 months: 95.1% *vs.* 80.1%, 36 months: 90.1% *vs.* 70.5%, 60 months: 77.5% *vs.* 53.2%). The no recurrence rates of negative infection prediction group were also higher (24 months: 73.1% *vs.* 69.6%, 36 months: 67.3% *vs.* 65.0%, 60 months: 62.5% *vs.* 33.6%) (Supplementary Table 5).

### Radscore of different CK7/CK19 expression

Radscore between different expression of CK7/CK19 was compared. There were 28 cases of positive CK7 expression (21.0%) and 106 cases of negative CK7 expression (79.0%). There were 17 cases (12.7%) in the CK19 positive expression group and 117 cases (87.3%) in the negative group. Radscore in the CK7 negative group and CK19 negative group was both significantly higher than in the positive group (Supplementary Table 6).

### CK7 prediction model

Clinical factors of positive and negative CK7 expression were compared and there was no significant difference. Also, there is significant correlation between liver fluke infection and CK7 positive expression (Table 3).

**Table 3:** Clinical parameters of CK7 positive and negative expression groups.

	CK7 positive (n = 28)	CK7 negative (n = 106)	*P* values
Liver fluke			0.001
Positive	19	36	
Negative	9	70	
Gender			0.062
Male	28	90	
Female	0	16	
Age (years)			0.722
> 50	14	49	
≤ 50	14	57	
Hypertension			0.169
Yes	4	5	
No	24	101	
Diabetes			0.101
Yes	4	4	
No	24	102	
Total bilirubin (μmol/L)			0.264
> 20.5	3	21	
≤ 20.5	25	85	
Direct bilirubin (μmol/L)			1.000
> 6.8	5	18	
≤ 6.8	23	88	
ALT (U/L)			0.262
> 45	13	37	
≤ 45	15	69	
AST (U/L)			0.818
> 40	12	48	
≤ 40	16	58	
HBV infection			0.166
Yes	21	93	
No	7	13	
AFP (ug/L)			0.048
≥ 400	5	40	
< 400	23	66	
Maximum tumor diameter (cm)			0.135
< 3	14	33	
3 ~ 5	8	33	
> 5	6	40	
BCLC stage			0.497
A	21	68	
B	5	30	
C	2	8	

*ALT* alanine aminotransferase, *AST* aspartate aminotransferase, *HBV* hepatitis B virus, *BCLC* Barcelona clinic liver cancer.

ROC curve of Radscore predicting CK7 positive expression was plotted (Supplementary Fig. 1A). The AUC was 0.687 (95% CI 0.573–0.790). Cutoff value was 62.967 according to the Youden index and all patients were divided into high risk (n = 83) and low risk groups (n = 51) of CK7 positive expression. Radscores between the two groups were significantly different (Supplementary Fig. 1B).

**Figure 8:**
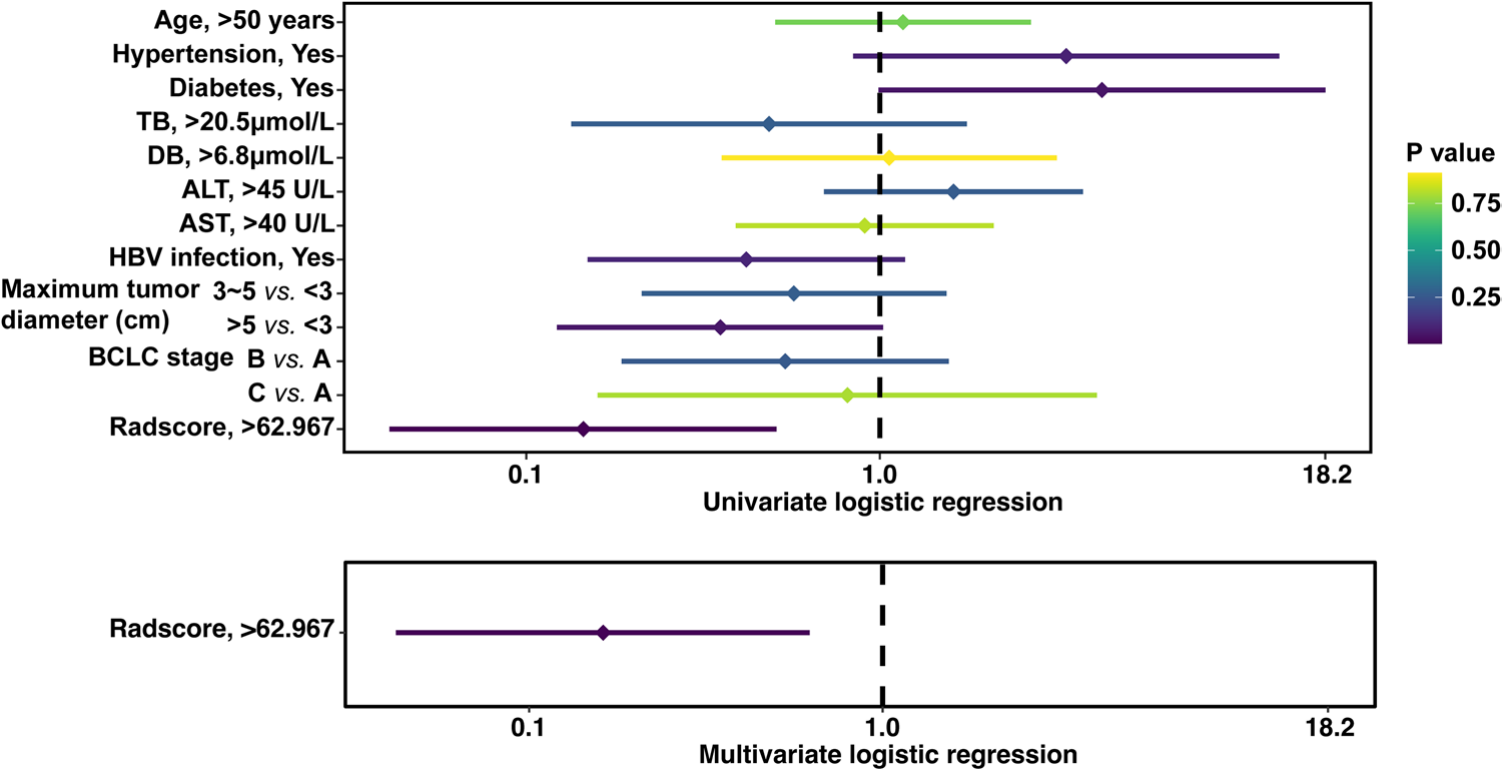
Univariate and multivariate logistic regression of clinical parameters and radiomics score for predicting CK7 expression.

Radscore and clinical parameters were included in univariate logistic regression to detect if they were predictors of positive CK7 expression. Radscore was an independent predictor for CK7 positive expression (*P* = 0.008, OR 0.162, 95 CI% 0.042–0.622) (Fig. 8) (Supplementary Table 7).

### Survival analysis of different CK7 expression and its prediction groups

**Figure 9:**
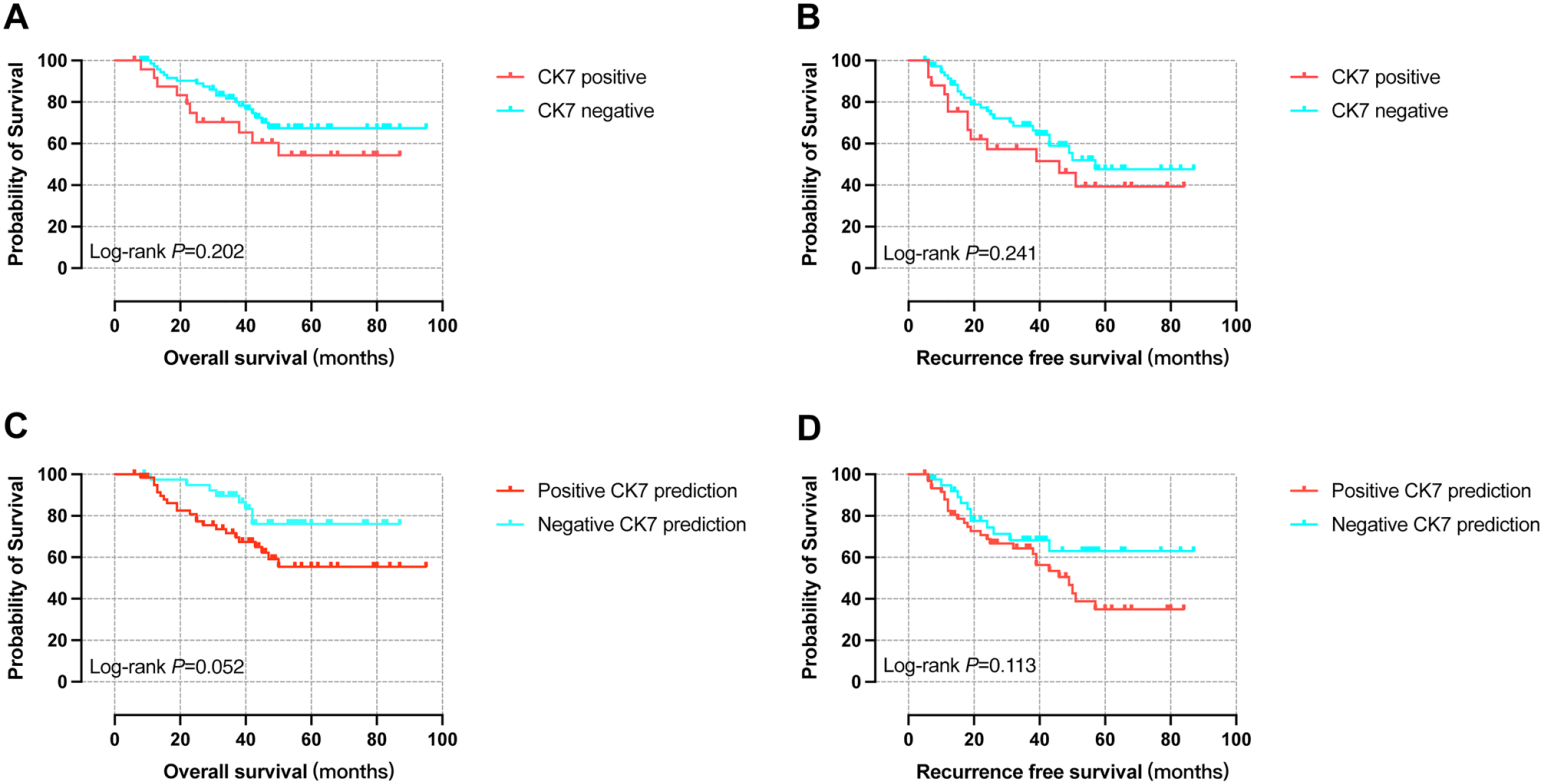
Kaplan–Meier survival analysis of different CK7 expression and its prediction groups. (**A**) OS curves of positive and negative CK7 expression, (**B**) RFS curves of positive and negative CK7 expression, (**C**) OS curves of positive and negative CK7 expression prediction, (**D**) RFS curves of positive and negative CK7 expression prediction. *OS* overall survival, *RFS* recurrence-free survival.

Compared with the negative CK7 expression group, OS of the positive group was poorer (P = 0.202) and the same was RFS (P = 0.241), while not statistically significant enough. In the positive CK7 prediction group, OS was also poorer than negative prediction group (P = 0.052), which was very close to being statistically significant and RFS was not good enough (P = 0.113) (Fig. 9).

Higher survival rates were observed in the negative CK7 expression group (24 months: 91.4% *vs.* 78.9%, 36 months: 85.9% *vs.* 68.4%, 60 months: 72.0% *vs.* 52.5%). Also, no recurrence rates of negative CK7 expression group were higher (24-months: 80.2% *vs.* 57.9%, 36 months: 70.8% *vs.* 54.7%, 60 months: 53.4% *vs.* 36.3%). The survival rates of negative CK7 expression group exceeded those of positive prediction group (24 months: 95.1% *vs.* 80.1%, 36 months: 90.1% *vs.* 70.5%, 60 months: 77.5% *vs.* 53.2%). Similarly, the no recurrence rates of negative CK7 expression group were higher (24 months: 73.1% *vs.* 69.6%, 36 months: 67.3% *vs.* 65.0%, 60 months: 62.5% *vs.* 33.6%) (Supplementary Table 8).

### CK19 prediction model

Clinical factors of different CK19 expression were compared. Age was significantly different between the two groups. The correlation between liver fluke infection and CK19 positive expression reached the borderline of significance (Table 4).

**Table 4:** Clinical parameters of CK19 positive and negative expression groups.

	CK19 Positive (n = 17)	CK19 Negative (n = 117)	*P* values
Liver fluke			0.111
Positive	10	45	
Negative	7	72	
Gender			1.000
Male	10	45	
Female	7	72	
Age (years)			0.038
> 50	15	103	
≤ 50	2	14	
Hypertension			1.000
Yes	4	59	
No	13	58	
Diabetes			1.000
Yes	1	8	
No	16	109	
Total bilirubin (μmol/L)			0.296
> 20.5	1	7	
≤ 20.5	16	110	
Direct bilirubin (μmol/L)			0.774
> 6.8	1	23	
≤ 6.8	16	94	
ALT (U/L)			0.374
> 45	2	21	
≤ 45	15	96	
AST (U/L)			0.469
> 40	8	42	
≤ 40	9	75	
HBV infection			0.450
Yes	9	51	
No	8	66	
AFP (ug/L)			0.348
≥ 400	4	41	
< 400	13	76	
Maximum tumor diameter (cm)			0.993
< 3	16	98	
3 ~ 5	1	19	
> 5	6	41	
BCLC stage			0.059
A	5	36	
B	6	40	
C	7	82	

*ALT* alanine aminotransferase, *AST* aspartate aminotransferase, *HBV* hepatitis B virus, *BCLC* Barcelona clinic liver cancer.

ROC curve of Radscore predicting CK19 positive expression was plotted (Supplementary Fig. 2A). AUC of the curve was 0.648 (95% CI 0.485–0.811). The cut-off value was 39.428. There were 61 cases in the high risk group of positive CK19 expression and 73 cases in the low risk group. The difference of Radscore between the two groups was significant (Supplementary Fig. 2B).

**Figure 10:**
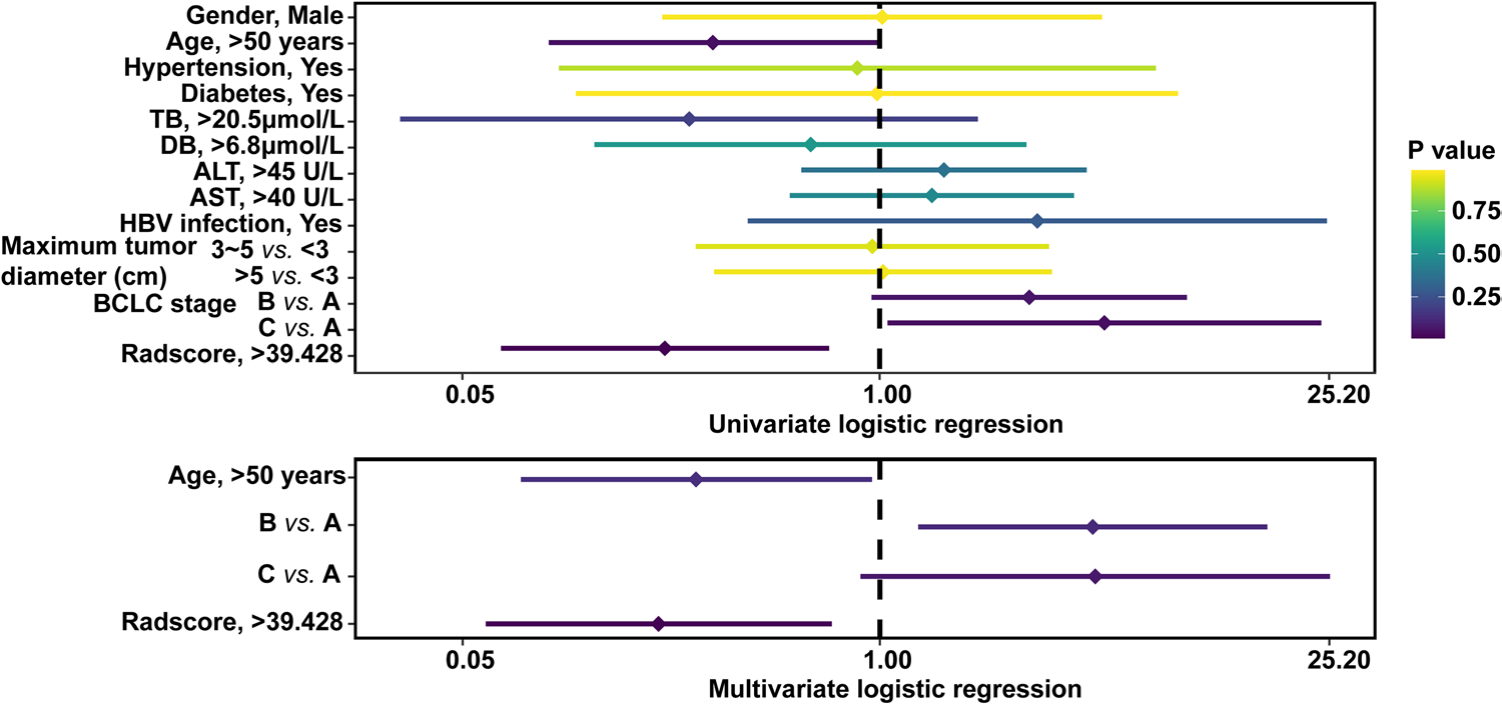
Univariate and multivariate logistic regression of clinical parameters and radiomics score for predicting CK19 expression.

Univariate logistic regression including Radscore and all clinical factors was carried out. Age and BCLC stages were only significant predictors of positive CK19 expression. Radscore was an independent predictor by multivariate logistic regression (Fig. 10) (Supplementary Table 9).

### Survival analysis of different CK19 expression and its prediction groups

**Figure 11:**
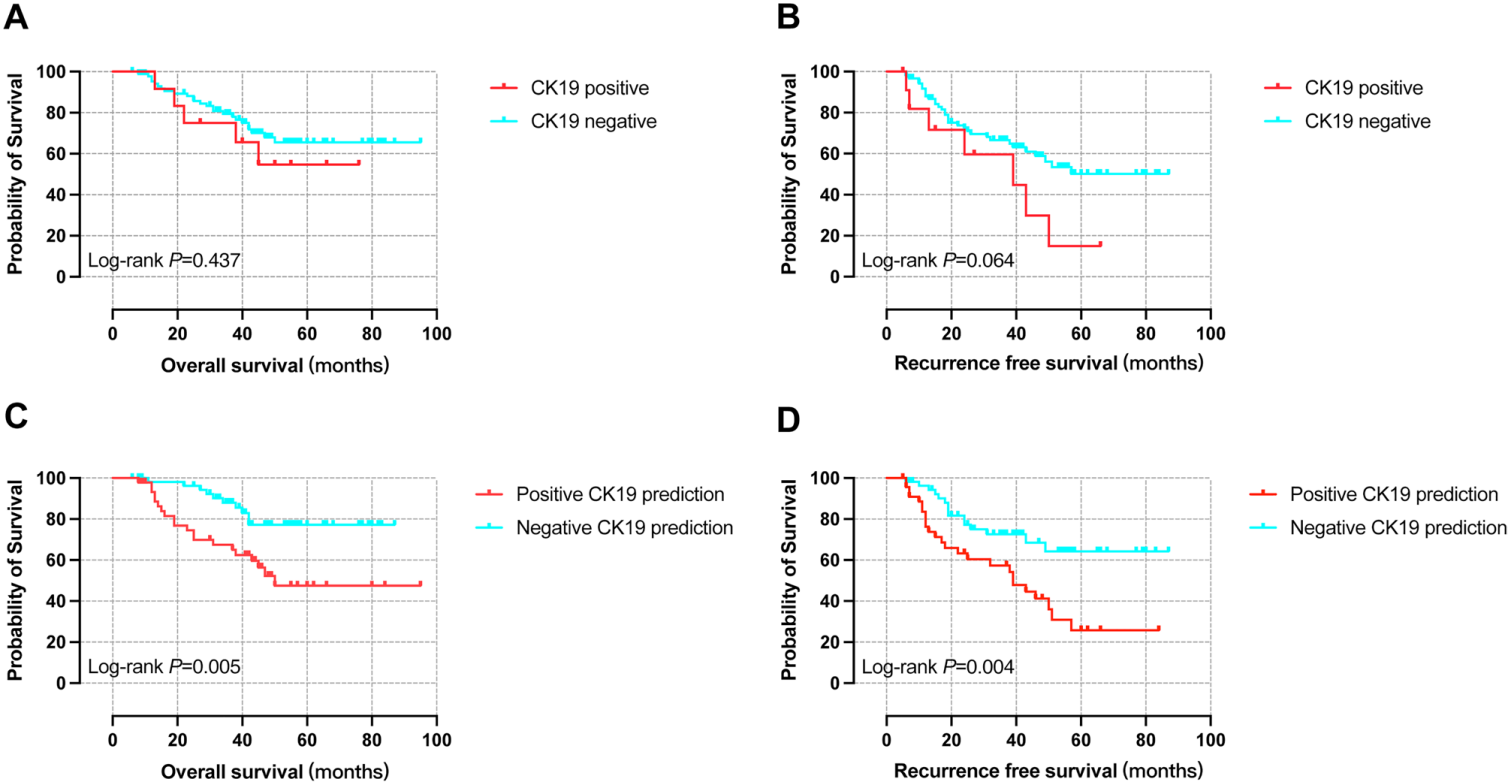
Kaplan–Meier survival analysis of different CK19 expression and its prediction groups. (**A**) OS curves of positive and negative CK19 expression, (**B**) RFS curves of positive and negative CK19 expression, (**C**) OS curves of positive and negative CK19 expression prediction, (**D**) RFS curves of positive and negative CK19 expression prediction. *OS* overall survival, *RFS* recurrence-free survival.

CK19 negative expression group harbored better OS (P = 0.437) and better RFS (P = 0.064) as well, though not reaching statistically significant. However, in the survival analysis of different CK19 expression prediction groups, OS and RFS of the negative group were significantly better (p = 0.005 and 0.004, respectively) (Fig. 11).

The survival rates of negative CK19 expression group were higher compared with the positive group (24 months: 88.1% *vs.* 75.0%, 36 months: 79.4% *vs.* 65.6%, 60 months: 65.5% *vs.* 54.7%) and the same were the no recurrence rates (24 months: 72.4% *vs.* 59.7%, 36 months: 66.6% *vs.* 44.7%, 60 months: 50.1% *vs.* 14.9%). Comparing RFS of positive and negative CK19 expression prediction groups, we found that for the negative prediction group, the survival rates (24 months: 96.2% *vs.* 74.5%, 36 months: 88.0% *vs.* 67.5%, 60 months: 77.2% *vs.* 47.5%) and no recurrence rates (24 months: 81.7% *vs.* 63.3%, 36 months: 72.6% *vs.* 57.4%, 60 months: 64.3% *vs.* 25.8%) were both higher (Supplementary Table 10).

## Discussion

Due to the diversity of dietary sources and eating habits, the incidence of liver fluke infection remains high. There are around 15 million cases of *Clonorchis sinensis* infection worldwide and 13 million cases in China^37^. At the same time, China is a country with high incidence of liver cancer, which is located within Asia and sub-Saharan Africa, the region with the highest incidence of liver cancer worldwide^5^. Liver cancer is the fifth leading cause of years of life lost in the Chinese population^38^.

Being in an area with high incidence of both liver fluke infection and liver cancer, the features of HCC with this epidemic background are worth discussing. It could contribute to the treatment of HCC patients in China and other regions with high incidence of liver fluke infection. The infection of liver fluke could interfere liver function to some extent. A study investigated liver enzyme levels of patients with *Fasciola hepatica* infection and ALT and AST levels both elevated in positive infection patients^39^. It was probably because of the secretion of serine protein kinase inhibitors form the parasite, which could protect from attacks of the host through a series of biological processes such as digestion, coagulation, inflammation and immune response^40^.

Studies of tumor prediction and further evaluation of its biological behaviors by radiomics have been carried out. Radiomics features were extracted from 46 HCC patients who underwent preoperative enhanced MRI and applied to classify tumor differentiation. Compared to tumors with high and moderate differentiation, the intensity of MRI images was lower and inconsistency of the features was higher in poorly differentiated tumors, compared to tumors with high and moderate differentiation. The inconsistency of the features could suggest tumor heterogeneity and aggressiveness^41^. Ji et al. analyzed radiomics features in the portal phase of enhanced CT in 177 biliary tract cancer patients. A nomogram was constructed to estimate the risk of lymph node metastasis and it showed satisfactory efficiency both in training and validation groups^42^.

There are certain studies about the imaging characteristics of liver lesions associated with liver fluke infection. Patutong et al. scanned the liver of hamsters with positive or negative infection of *Opisthorchis viverrine* using Fourier infrared spectroscopy. Scans in the positive infection group were performed in 1 month, 2 months, 3 months and 6 months post-infection. Principal component analysis of images could identify normal liver tissue, early-stage cholangiocarcinoma and cholangiocarcinoma^43^. In another study on hamsters infected with *Opisthorchis felineus*, after 8 weeks of infection, MRI images of the hamster liver could indicate the degree of tissue damage^44^. In our research, 5636 radiomics features were extracted from all four phases of MRI images. After data dimension reduction by VarianceThreshold, SelectKBest and LASSO, 11 features were included in the combined radiomics model. The combined model showed better performance than any of AP, PVP, DP and HBP models for predicting liver fluke infection in HCC patients. Interestingly, we found that both OS and RFS of negative liver fluke infection group were significantly better than the positive group. What’s more, negative infection prediction group by the radiomics model had better OS and RFS, suggesting more possible practical value of the model.

Various immunohistochemical indicators are related to HCC and CK7 is an important one. CK7 is a marker of bile duct epithelial cells and can also indicate HCC cells have properties of hepatic progenitor cells. In an animal study, HCC in mice was induced by different doses of diethylnitrosamine (DEN). The expression level of CK7 was associated with tumor progression and prognosis^45^. For patients with preliminary diagnosis of early-stage HCC by enhanced MRI, when typical changes of HCC were hardly to be found in pathology of ultrasound-guided biopsy, CK7 and Victorian Blue staining, which could suggest bile duct response and terminal portal duct invasion separately, could contribute to the diagnosis^46^. CK19, another valuable immunohistochemical indicator, is a kind of intermediate filament and suggests poor prognosis^47^. CK19-positive HCC possesses characteristics of hepatic progenitor cells and tends to be more active in epithelial-mesenchymal transition and angiogenesis^28, 29^. The expression of CK19 needs to be confirmed by pathological examination, however, there are several studies on assessing its expression by imaging prior to surgery. A total of 84 HCC patients who underwent gadoxetic acid-enhanced MRI were included in a study. It turned out that atypical images like weaker intensification in artery phase and gradual intensification in portal vein and delayed phases could suggest positive CK19 expression^48^. Choi et al. manifested that for preoperative enhanced MRI images of HCC patients, specific signals in hepatobiliary phase could be predictors of positive CK19 expression^49^. Kawai et al. conducted a study that included 98 HCC patients who underwent preoperative Positron Emission Tomography (PET)-CT. The standardized uptake values (SUV) showed good effects in suggesting CK19 expression and signals of CK19-positive tumors were significantly higher^50^. Both CT and MRI are imaging examinations throughout the course of HCC diagnosis and treatment. With 18F-fluorodeoxyglucose (18F-FDG), PET-CT could reflect the level of metabolic activity within and around the liver tumor^51^. It is also capable of detecting distant metastasis of liver cancer^52^. However, the cost of PET-CT is relatively high, may leading to excessive financial burden^53^. MRI harbors high resolution, enabling clear visualization of anatomical structure^51^. Also, the cost of MRI is more affordable^53^. However, MRI requires long acquisition time and some patients may not complete the examination due to discomfort or an inability to tolerate the procedure^54^. Another study on enhanced MRI of HCC showed the enhancement ratio and enhanced signal to noise signal ratio could indicate the expression of CK7 and CK19. The enhancement ratio was significantly lower in CK19-positve group compared to negative group, while the ratio was higher in CK7-positive group, but not significantly^55^. In this study, firstly, liver fluke infection was found to be significantly associated with CK7/19 expression. Then, Radscore could be applied to predict CK7 and CK19 expression with satisfactory efficiency. After setting specific cut-off value, it could effectively distinguish high-risk group of positive expression from the low-risk group. Survival analyses of different CK7 expression groups and CK7 prediction groups showed that HCC with positive CK7 expression tended to lead to poorer OS and RFS and our radiomics model could distinguish patients with miserable survival when predicting CK7 expression at the same time. What’s more, HCC with positive CK19 may cause worse OS and RFS, though showing an unsignificant trend, and the radiomics model exhibited significant efficiency in survival analysis while predicting CK19 expression.

However, there were still some limitations in our research. First, this study was carried in a single center and multi-center data may be needed to further validate the results. Also, the number of patients enrolled in the study was relatively small due to strict inclusion and exclusion criteria. Further cell and animal experiments are required to validate the conclusions in this paper, which are aimed at exploring the biological behavior of HCC cells with different CK7/CK19 expression status with or without intervention with liver fluke components and metabolites. Additionally, animal experiments should be conducted.

In conclusion, a radiomics model based on gadoxetic acid-enhanced MRI images was established to predict liver fluke infection among treatment naive HCC patients. This model could also be used to indicate CK7 and CK19 expression in the HCC tumor tissue with different backgrounds of liver fluke infection prior to surgery and a certain degree of "virtual biopsy" of the tumor tissue could be performed. Also, the model could predict OS and RFS of HCC patients when predicting liver fluke infection, CK7 and CK19 expression, further broadening the application of the model. This study has expanded the practical value of gadoxetic acid-enhanced MRI to some extent. Moreover, it can contribute to the assessment of HCC patients in liver fluke epidemic areas around the world.

### Supplementary Information

Supplementary Legends.

Supplementary Figure S1.

Supplementary Figure S2.

Supplementary Tables.

## Data Availability

All the data in this research are available under reasonable request by contacting the corresponding author.
