# Supplementary material for: A radiomics model based on magnetic resonance imaging to predict cytokeratin 7/19 expression and liver fluke infection of hepatocellular carcinoma
Source: Sci Rep. 2023 Oct 16;13:17553. doi: 10.1038/s41598-023-44773-5 (PMC10579381; doi:10.1038/s41598-023-44773-5)
Supplement: Supplementary file 1 — Supplementary Legends. [file 41598_2023_44773_MOESM1_ESM.docx]

Supplementary Figure1 ROC curve of Radscore predicting CK7 positive expression (A) and Radscores of high-risk and low-risk groups of CK7 positive expression (B)

ROC, receiver operating characteristic

Supplementary Figure2 ROC curve of Radscore predicting CK19 positive expression (A) and Radscores of high-risk and low-risk groups of CK19 positive expression (B)

ROC, receiver operating characteristic
