# Supplementary figures and images for: A radiomics model based on magnetic resonance imaging to predict cytokeratin 7/19 expression and liver fluke infection of hepatocellular carcinoma
Source: Sci Rep. 2023 Oct 16;13:17553. doi: 10.1038/s41598-023-44773-5 (PMC10579381; doi:10.1038/s41598-023-44773-5)

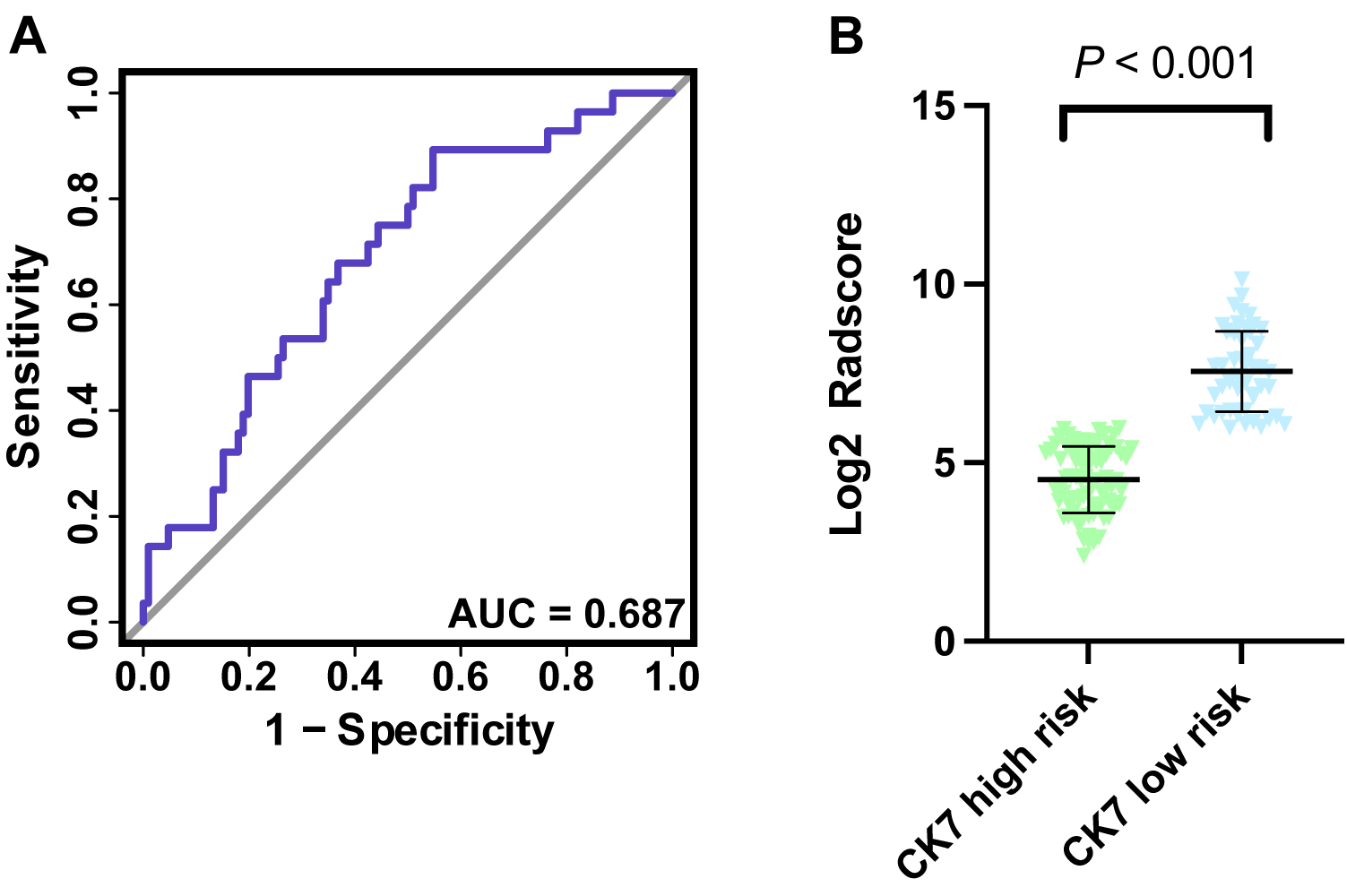

Supplement: Supplementary file 2 — Supplementary Figure S1. [file 41598_2023_44773_MOESM2_ESM.tif]

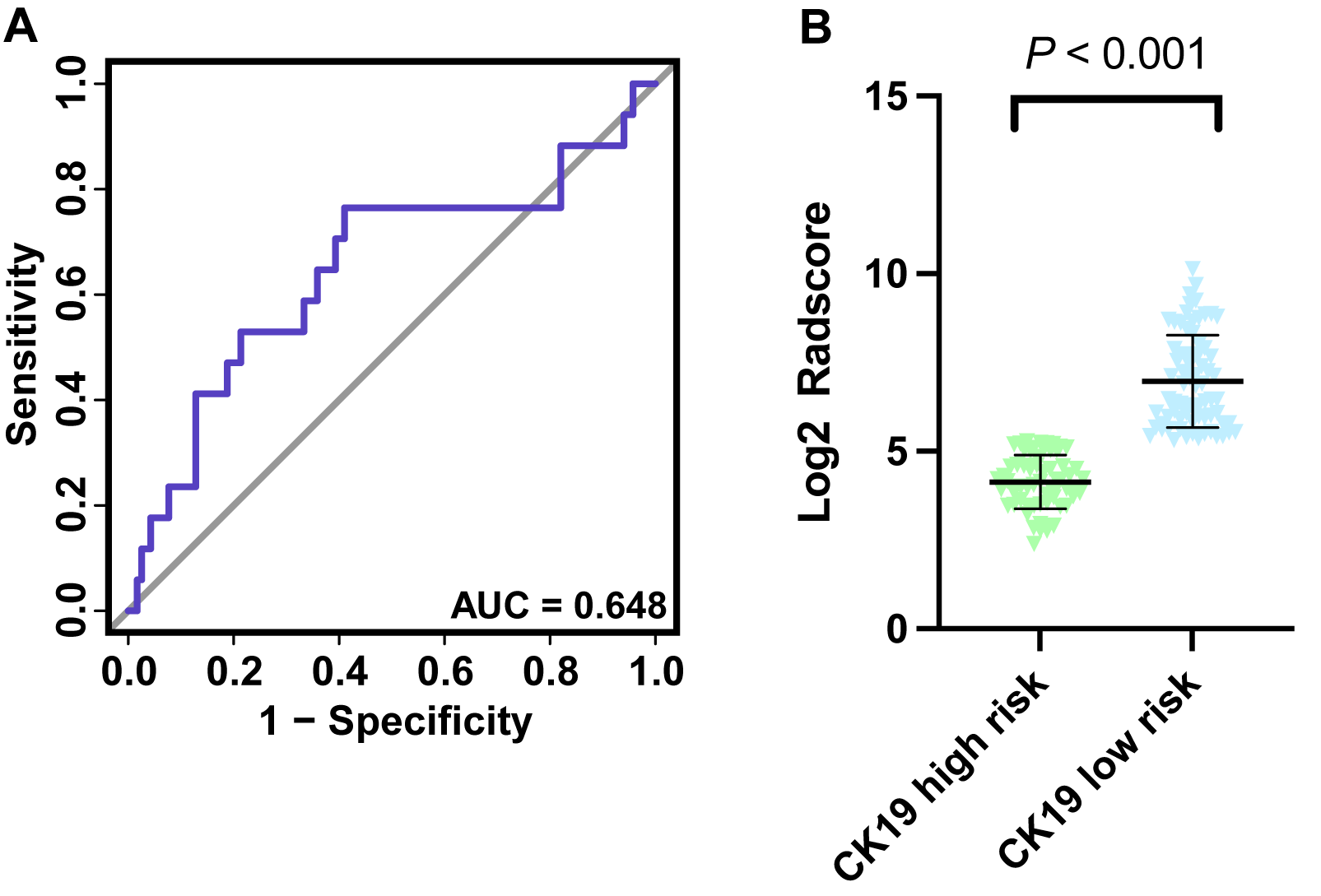

Supplement: Supplementary file 3 — Supplementary Figure S2. [file 41598_2023_44773_MOESM3_ESM.tif]
