# Supplementary material for: A radiomics model based on magnetic resonance imaging to predict cytokeratin 7/19 expression and liver fluke infection of hepatocellular carcinoma
Source: Sci Rep. 2023 Oct 16;13:17553. doi: 10.1038/s41598-023-44773-5 (PMC10579381; doi:10.1038/s41598-023-44773-5)
Supplement: Supplementary file 4 — Supplementary Tables. [file 41598_2023_44773_MOESM4_ESM.docx]

Supplementary Table 1 Features in the combined model and corresponding regression coefficients

| No. | Features | Regression coefficients |
| --- | --- | --- |
| 1 | 4_square_firstorder_Maximum | 0.15263 |
| 2 | 1_wavelet-LLH_firstorder_Skewnes | 0.11552 |
| 3 | 2_square_firstorder_90Percentile | 0.09091 |
| 4 | 2_wavelet_HLH_gldm_LargeDependence  LowGrayLevelEmphasis | 0.03570 |
| 5 | 2_original_firstorder_10Percentile | 0.03004 |
| 6 | 4_square_firstorder_RootMeanSquared | 0.02610 |
| 7 | 4_square_firstorder_Energy | 0.00987 |
| 8 | 4_original_firstorder_10Percentile | 0.00507 |
| 9 | 4_logarithm_firstorder_Maximum | -0.00147 |
| 10 | 2_squareroot_firstorder_90Percentile | -0.02456 |
| 11 | 3_squareroot_firstorder_RootMeanSquared | -0.10749 |

Supplementary Table 2 Support vector machines and K-nearest neighbor classifiers to evaluate the performance of each single-phase model and combined model

| Groups | Classifires | Models | AUC (95%CI) | Sensitivity | Specifiticity |
| --- | --- | --- | --- | --- | --- |
| Training group | SVM | AP | 0.851 (0.780-0.922) | 0.70 | 0.69 |
|  |  | PVP | 0.786 (0.697-0.876) | 0.77 | 0.76 |
|  |  | DP | 0.747 (0.651-0.843) | 0.76 | 0.75 |
|  |  | HBP | 0.606 (0.496-0.717) | 0.61 | 0.60 |
|  |  | Combined | 0.893 (0.836-0.951) | 0.77 | 0.77 |
|  | KNN | AP | 0.779 (0.693-0.875) | 0.64 | 0.83 |
|  |  | PVP | 0.844 (0.765-0.937) | 0.73 | 0.84 |
|  |  | DP | 0.844 (0.762-0.934) | 0.73 | 0.84 |
|  |  | HBP | 0.714 (0.626-0.817) | 0.52 | 0.73 |
|  |  | Combined | 0.811 (0.735-0.903) | 0.61 | 0.81 |
| Validation group | SVM | AP | 0.576 (0.339-0.812) | 0.55 | 0.53 |
|  |  | PVP | 0.688 (0.459-0.916) | 0.73 | 0.63 |
|  |  | DP | 0.830 (0.635-1.000) | 0.64 | 0.81 |
|  |  | HBP | 0.625 (0.405-0.845) | 0.64 | 0.56 |
|  |  | Combined | 0.919 (0.871-0.967) | 0.82 | 0.93 |
|  | KNN | AP | 0.539 (0.353-0.736) | 0.36 | 0.67 |
|  |  | PVP | 0.602 (0.413-0.805) | 0.45 | 0.69 |
|  |  | DP | 0.793 (0.628-0.958) | 0.66 | 0.67 |
|  |  | HBP | 0.500 (0.300-0.700) | 0.36 | 0.50 |
|  |  | Combined | 0.924 (0.795-1.000) | 0.73 | 1.00 |
| Whole group | SVM | AP | 0.800 (0.726-0.875) | 0.91 | 0.55 |
|  |  | PVP | 0.766 (0.682-0.849) | 0.73 | 0.76 |
|  |  | DP | 0.725 (0.637-0.812) | 0.60 | 0.82 |
|  |  | HBP | 0.613 (0.516-0.711) | 0.56 | 0.69 |
|  |  | Combined | 0.899 (0.849-0.949) | 0.75 | 0.93 |
|  | KNN | AP | 0.733 (0.652-0.815) | 0.80 | 0.58 |
|  |  | PVP | 0.794 (0.720-0.868) | 0.81 | 0.67 |
|  |  | DP | 0.758 (0.681-0.836) | 0.81 | 0.58 |
|  |  | HBP | 0.672 (0.587-0.757) | 0.43 | 0.91 |
|  |  | Combined | 0.830 (0.766-0.895) | 0.58 | 0.91 |

AUC, area under curve; CI, confidence interval; SVM, support vector machines; KNN, K-nearest neighbor; AP, arterial phase; PVP, portal venous phase; DP, delayed phase; HBP, hepatobiliary phase

Supplementary Table 3 Comparison between the combined model and arterial phase, portal vein phase, delayed phase and hepatobiliary phase models in the training, validation and whole groups

| Groups | Model | Models | *P* values |
| --- | --- | --- | --- |
| Training | Combined | AP | 0.350 |
|  |  | PVP | 0.060 |
|  |  | DP | 0.010 |
|  |  | HBP | <0.001 |
| Validation | Combined | AP | 0.008 |
|  |  | PVP | 0.056 |
|  |  | DP | 0.362 |
|  |  | HBP | 0.014 |
| Whole | Combined | AP | 0.004 |
|  |  | PVP | 0.002 |
|  |  | DP | 0.002 |
|  |  | HBP | <0.001 |

AP, arterial phase; PVP, portal venous phase; DP, delayed phase; HBP, hepatobiliary phase

Supplementary Table 4 Univariate and multivariate logistic regression of clinical parameters and radiomics score for predicting liver fluke infection

|  | Univariate |  | Multivariate |  |  |
| --- | --- | --- | --- | --- | --- |
|  | *P* values | OR | *P* values | OR | 95%CI |
| Gender, Male | 0.173 | 2.284 |  |  |  |
| Age, >50 years | 0.513 | 0.794 |  |  |  |
| Hypertension, Yes | 0.830 | 1.161 |  |  |  |
| Diabetes, Yes | 0.597 | 1.471 |  |  |  |
| TB, >20.5μmol/L | 0.946 | 1.032 |  |  |  |
| DB, >6.8μmol/L | 0.236 | 1.725 |  |  |  |
| ALT, >45 U/L | 0.020 | 2.348 | 0.090 | 0.407 | 0.144-1.152 |
| AST, >40 U/L | 0.124 | 1.727 |  |  |  |
| HBV infection, Yes | 0.380 | 0.652 |  |  |  |
| Maximum tumor diameter (cm) |  |  |  |  |  |
| 3～5 *vs.* <3 | 0.233 | 1.681 |  |  |  |
| >5 *vs.* <3 | 0.768 | 1.134 |  |  |  |
| BCLC stage |  |  |  |  |  |
| B *vs.* A | 0.091 | 1.981 |  |  |  |
| C *vs.* A | 0.131 | 2.806 |  |  |  |
| Radscore, >60.964 | 0.001 | 0.259 | 0.001 | 0.143 | 0.054-0.379 |

OR, odds ratio; TB, total bilirubin; DB, direct bilirubin; ALT, alanine aminotransferase; AST, aspartate aminotransferase; HBV, hepatitis B virus; BCLC, Barcelona clinic liver cancer

Supplementary Table 5 Kaplan-Meier survival analyses of positive and negative liver fluke infection groups and liver fluke infection positive and negative prediction groups

|  |  |  | | Overall survival rate | | | | | |  | | | Recurrence free survival rate | | | | | |  | | | | |  |  |
| --- | --- | --- | --- | --- | --- | --- | --- | --- | --- | --- | --- | --- | --- | --- | --- | --- | --- | --- | --- | --- | --- | --- | --- | --- | --- |
|  |  | No. | | 24 months | | 36 months | | 60 months | | | *P* values | | 24 months | | | 36 months | 60 months | | | *P* values | | | | |  |
| Liver fluke | Positive | | 38 | | 78.9 | | 68.4 | | 52.5 | | | 0.039 | | 57.9 | 54.7 | | | 36.3 | | | 0.026 | |  |  |  |
|  | Negative | | 65 | | 91.4 | | 85.9 | | 72.0 | | |  | | 80.2 | 70.8 | | | 53.4 | | |  | |  |  |  |
| Liver fluke | Positive infection prediction | | 61 | | 80.1 | | 70.5 | | 53.2 | | | 0.024 | | 69.6 | 65.0 | | | 33.6 | | | 0.042 | |  |  |  |
|  | Negative infection prediction | | 42 | | 95.1 | | 90.1 | | 77.5 | | |  | | 73.1 | 67.3 | | | 62.5 | | | |  | | | |

Supplementary Table 6 Comparison of Radscore between different expression of CK7/CK19

|  |  | Number | Radscore | *P* values |
| --- | --- | --- | --- | --- |
| CK7 | Positive | 28 | 33.405 (15.221;49.708) | 0.002 |
|  | Negative | 106 | 50.019 (22.605;143.561) |  |
| CK19 | Positive | 17 | 18.622 (12.4223;112.834) | 0.049 |
|  | Negative | 117 | 46.808 (21.962;126.826) |  |

Notes: Radscore was showed as Median (*P*_25_; *P*_75_).

Supplementary Table 7 Univariate and multivariate logistic regression of radiomics score and clinical parameters for predicting CK7 positive expression

|  | Univariate |  | Multivariate |  |  |
| --- | --- | --- | --- | --- | --- |
|  | *P* values | OR | *P* values | OR | 95%CI |
| Gender, Male | 0.998 |  |  |  |  |
| Age, >50 years | 0.722 | 1.163 |  |  |  |
| Hypertension, Yes | 0.087 | 3.367 |  |  |  |
| Diabetes, Yes | 0.051 | 4.250 |  |  |  |
| TB, >20.5μmol/L | 0.272 | 0.486 |  |  |  |
| DB, >6.8μmol/L | 0.913 | 1.063 |  |  |  |
| ALT, >45 U/L | 0.264 | 1.616 |  |  |  |
| AST, >40 U/L | 0.818 | 0.906 |  |  |  |
| HBV infection, Yes | 0.099 | 0.419 |  |  |  |
| Maximum tumor diameter (cm) |  |  |  |  |  |
| 3～5 *vs.* <3 | 0.270 | 0.571 |  |  |  |
| >5 *vs.* <3 | 0.055 | 0.354 |  |  |  |
| BCLC stage |  |  |  |  |  |
| B *vs.* A | 0.257 | 0.540 |  |  |  |
| C *vs.* A | 0.799 | 0.810 |  |  |  |
| Radscore, > 62.967 | 0.003 | 0.145 | 0.008 | 0.162 | 0.042-0.622 |

OR, odds ratio; TB, total bilirubin; DB, direct bilirubin; ALT, alanine aminotransferase; AST, aspartate aminotransferase; HBV, hepatitis B virus; BCLC, Barcelona clinic liver cancer

Supplementary Table 8 Kaplan-Meier survival analyses of CK7 positive and negative expression groups and CK7 positive and negative prediction groups

|  |  |  | Overall survival rate | | |  | Recurrence free survival rate | | | | | |  | |
| --- | --- | --- | --- | --- | --- | --- | --- | --- | --- | --- | --- | --- | --- | --- |
|  |  | No. | 24 months | 36 months | 60 months | *P* values | 24 months | | 36 months | | 60 months | | *P* values | |
| CK7 | Positive | 25 | 74.8 | 70.4 | 54.3 | 0.202 | 57.3 | 51.6 | | 39.3 | | | | 0.241 |
|  | Negative | 78 | 90.3 | 81.6 | 67.5 |  | 75.7 | 68.6 | | 47.7 | | | |  |
| CK7 | Positive expression prediction | 63 | 80.8 | 71.6 | 55.4 | 0.052 | 68.8 | 64.3 | | 35.0 | | | | 0.113 |
|  | Negative expression prediction | 40 | 94.9 | 89.6 | 76.1 |  | 74.5 | 68.3 | | 63.0 | |  | | |

Supplementary Table 9 Univariate and multivariate logistic regression of radiomics score and clinical parameters for predicting CK19 positive expression

|  | Univariate | |  | | Multivariate | |  | |  |
| --- | --- | --- | --- | --- | --- | --- | --- | --- | --- |
|  | *P* values | | OR | | *P* values | | OR | | 95%CI |
| Gender, Male | 0.981 | 1.019 | |  | |  | |  | |
| Age, >50 years | 0.047 | 0.302 | | 0.141 | | 0.267 | | 0.076-0.945 | |
| Hypertension, Yes | 0.883 | 0.852 | |  | |  | |  | |
| Diabetes, Yes | 0.987 | 0.982 | |  | |  | |  | |
| TB, >20.5μmol/L | 0.197 | 0.255 | |  | |  | |  | |
| DB, >6.8μmol/L | 0.531 | 0.610 | |  | |  | |  | |
| ALT, >45 U/L | 0.377 | 1.587 | |  | |  | |  | |
| AST, >40 U/L | 0.470 | 1.456 | |  | |  | |  | |
| HBV infection, Yes | 0.286 | 3.102 | |  | |  | |  | |
| Maximum tumor diameter (cm) |  |  | |  | |  | |  | |
| 3～5 *vs.* <3 | 0.936 | 0.949 | |  | |  | |  | |
| >5 *vs.* <3 | 0.968 | 1.025 | |  | |  | |  | |
| BCLC stage |  |  | |  | |  | |  | |
| B *vs.* A | 0.063 | 2.929 | | 0.117 | | 4.609 | | 1.316-16.148 | |
| C *vs.* A | 0.042 | 5.020 | | 0.072 | | 4.694 | | 0.870-25.31 | |
| Radscore, >39.428 | 0.010 | 0.214 | | 0.012 | | 0.204 | | 0.059-0.708 | |

OR, odds ratio; TB, total bilirubin; DB, direct bilirubin; ALT, alanine aminotransferase; AST, aspartate aminotransferase; HBV, hepatitis B virus; BCLC, Barcelona clinic liver cancer

Supplementary Table 10 Kaplan-Meier survival analyses of CK19 positive and negative expression groups and CK19 positive and negative prediction groups

|  |  |  | Overall survival rate | | |  | | Recurrence free survival rate | | | | | | |  | |
| --- | --- | --- | --- | --- | --- | --- | --- | --- | --- | --- | --- | --- | --- | --- | --- | --- |
|  |  | No. | 24 months | 36 months | 60 months | *P* values | | 24 months | | | | 36 months | 60 months | | *P* values | |
| CK19 | Positive | 12 | 75.0 | 65.6 | 54.7 | | 0.437 | 59.7 | | 44.7 | | | 14.9 | | | 0.064 |
|  | Negative | 91 | 88.1 | 79.4 | 65.5 | |  | 72.4 | | 66.6 | | | 50.1 | | |  |
| CK19 | Positive expression prediction | 46 | 74.5 | 67.5 | 47.5 | | 0.005 | 63.3 | | 57.4 | | | 25.8 | | | 0.004 |
|  | Negative expression prediction | 57 | 96.2 | 88.0 | 77.2 |  | | | 81.7 | | 72.6 | | | 64.3 | |  |
